# Hierarchical Cluster Analysis of Three-Dimensional Reconstructions of Unbiased Sampled Microglia Shows not Continuous Morphological Changes from Stage 1 to 2 after Multiple Dengue Infections in *Callithrix penicillata*

**DOI:** 10.3389/fnana.2016.00023

**Published:** 2016-03-22

**Authors:** Daniel G. Diniz, Geane O. Silva, Thaís B. Naves, Taiany N. Fernandes, Sanderson C. Araújo, José A. P. Diniz, Luis H. S. de Farias, Marcia C. K. Sosthenes, Cristovam G. Diniz, Daniel C. Anthony, Pedro F. da Costa Vasconcelos, Cristovam W. Picanço Diniz

**Affiliations:** ^1^Laboratório de Investigações em Neurodegeneração e Infecção, Instituto de Ciências Biológicas, Universidade Federal do Pará, Hospital Universitário João de Barros BarretoBelém, Brasil; ^2^Experimental Neuropathology Laboratory, Department of Pharmacology, University of OxfordOxford, UK; ^3^Curso de Graduação em Biologia, Universidade da AmazôniaBelém, Brasil; ^4^Departamento de Microscopia Eletrônica, Instituto Evandro ChagasBelém, Brasil; ^5^Instituto de Ciências Biológicas, Universidade Federal do ParáBelém, Brasil; ^6^Laboratório de Biologia Molecular e Ambiental, Instituto Federal de Educação Ciência e Tecnologia do Pará, Campus de Bragança, BragançaPará, Brasil; ^7^Departamento de Arbovirologia e Febres Hemorrágicas, Instituto Evandro ChagasAnanindeua, Brasil

**Keywords:** dengue virus infection, microglial morphological subtleties, hierarchical cluster analysis, dentate gyrus, *Callithrix penicillata*

## Abstract

It is known that microglial morphology and function are related, but few studies have explored the subtleties of microglial morphological changes in response to specific pathogens. In the present report we quantitated microglia morphological changes in a monkey model of dengue disease with virus CNS invasion. To mimic multiple infections that usually occur in endemic areas, where higher dengue infection incidence and abundant mosquito vectors carrying different serotypes coexist, subjects received once a week subcutaneous injections of DENV3 (genotype III)-infected culture supernatant followed 24 h later by an injection of anti-DENV2 antibody. Control animals received either weekly anti-DENV2 antibodies, or no injections. Brain sections were immunolabeled for DENV3 antigens and IBA-1. Random and systematic microglial samples were taken from the polymorphic layer of dentate gyrus for 3-D reconstructions, where we found intense immunostaining for TNFα and DENV3 virus antigens. We submitted all bi- or multimodal morphological parameters of microglia to hierarchical cluster analysis and found two major morphological phenotypes designated types I and II. Compared to type I (stage 1), type II microglia were more complex; displaying higher number of nodes, processes and trees and larger surface area and volumes (stage 2). Type II microglia were found only in infected monkeys, whereas type I microglia was found in both control and infected subjects. Hierarchical cluster analysis of morphological parameters of 3-D reconstructions of random and systematic selected samples in control and ADE dengue infected monkeys suggests that microglia morphological changes from stage 1 to stage 2 may not be continuous.

## Introduction

Microglia are often categorized as resting or activated based on a qualitative assessment of their morphology. However, it is now known that resting microglial cells are both motile and very active. It has also been noted that rounded, activated microglia are often less motile and that while “activated” microglia can release potentially damaging proteases and free radicals this is not always the case. In the disease free individual resting microglia are highly ramified and express very low levels of CD40, MHC class II, and B7-2. However, this morphology is just an extreme example of a dynamic process that appears to be a continuum of morphological changes. Indeed, microglial morphology may change in association with neuroprotective, proinflammatory, cytotoxic, immunoregulatory, and repair functions (Hanisch and Kettenmann, 2007; Benarroch, 2013; Miyamoto et al., 2013; Gomez-Nicola and Perry, 2015). A variety of different models have been proposed to characterize microglial morphological changes after brain damage (Beynon and Walker, 2012; Walker et al., 2014). With small variations, we have learned from these schemes that microglia may transition through at least four different morphological phenotypes after injury: ramified microglia (Stage 1), hyper-ramified reactive microglia (Stage 2), “reactive” state microglia (Stage 3), and finally, “phagocytic” microglia (Stage 4) (Davis et al., 1994; Streit et al., 1999; Stence et al., 2001). More recently, a novel classification following axotomy, based on hierarchical cluster analysis of 2D and 3D cell morphometric features has been proposed (Yamada and Jinno, 2013).

It has been described using chronic stress models, that microglial branching may represent the initial activation phase (Hinwood et al., 2013) which will develop into microglia with shorter and fewer branches at later stages of activation. When the activation process ends microglia may change to the homeostatic cell morphology returning to a non-activated state. Recently, microscopic three-dimensional reconstruction of microglia, followed by hierarchical cluster analysis of the morphometric features, was used to objectively describe and classify morphological changes under homeostatic and neuropathological conditions (Yamada and Jinno, 2013; Torres-Platas et al., 2014; Papageorgiou et al., 2015). However, none of the previous investigations quantified subtle microglial changes caused by virus infection in primates. The model of antibody-enhanced dengue disease (ADE) provides an opportunity to explore the virus-associated morphological changes using a stereological sampling approach.

Sequential dengue infections by different serotypes are usually associated with severe dengue, and this severity might be a result of antibody-dependent enhancement mechanism. Dengue virus infection of myeloid cells is facilitated by serotype cross-reactive antibodies through Fc receptors (Halstead, 2003; Ng et al., 2014). Anti-DENV antibodies injection enhance dengue virus infection and disease in mice (Balsitis et al., 2010; Diniz et al., 2012) and all anti-DENV antibodies enhanced infection at subneutralizing concentrations (Beltramello et al., 2010). Thus, cross-reactivity during acute primary and secondary infections (Friberg et al., 2011) may increase cytokine levels and viremia observed in severe disease (Boonnak et al., 2011; Ng et al., 2014).

In a previous report, we tested the ADE model in mice (Diniz et al., 2012, 2013) and translated ADE to *Callithrix penicillata* (the black-tufted marmoset) (Vasconcelos et al., 2016). The inflammatory response was characterized, both in the periphery and in the CNS, and marked changes in CNS pathology characterized by extensive microglial activation and TNFα immunolabeling was confirmed. In the present report we used stereological sampling approach, microscopic 3D reconstruction and hierarchical cluster analysis to classify reactive microglia from dentate gyrus of previous ADE study. Because we found frequent clusters of activated microglia and intense TNFα immunolabeling in the polymorphic layer of dentate gyrus of infected monkeys, we selected this layer as our target to investigate detailed microglial morphological changes. Microglia were classified according to previous descriptions of mouse encephalitis (de Sousa et al., 2015) and hypoglossal axotomy (Yamada and Jinno, 2013).

## Materials and methods

### Experimental procedures

Ethics Committee on Animal Research at Evandro Chagas Institute, Primate National Center (IEC-CENP) (protocol #0061/2009) and by the System Authorization and Information on Biodiversity-SISBIO of Chico Mendes, Institute for Biodiversity Conservation-ICMBio (protocol #22047-3), and the Institute of the Brazilian Environment-IBAMA, License Number 004-2013 for Wild Animal Transport and Ethics Committee on Animal Research at the Federal University of Para (CEPAE/UFPA 155-13) approved all experimental procedures. In this study, the viral sample of serotype DENV3 (ROR 3115) used was obtained from the Hemorrhagic Fever and Arbovirus Section at Evandro Chagas Institute. The authorization for its use was received through protocol #006031/2013-91. The animals used in this study were selected from the *C. penicillata* colony at the Centro Nacional de Primatas (CENP), located in Ananindeua, Pará, Brazil. Individuals used in the present report were negative in the hemagglutination inhibition assay test for 23 different types of arboviruses. Belém virus; Bussuquara virus; Cacipacore virus; Caraparu virus; Catu virus; DENV1, 2, 3, and 4; Eastern equine encephalitis virus; Guaroa virus; Icoaraci virus; Ilheus virus; Maguari virus; Mayaro virus; Mucambo virus; Oropouche virus; Rocio virus; St. Louis encephalitis virus; Tacaiuma virus; Utinga virus; Western equine encephalitis virus, and yellow fever virus were tested in the screening and all animals showed negative results in the hemagglutination.

### Housing conditions and experimental time line

All animals shared an enriched room (408 × 259 × 276 cm high) equipped with ropes, mirrors, cages, hammock, stairs, bridge, swings, cages, and toys. They were monitored 24 h a day using video camera. All of the animals had free access to water and were fed once or twice a day. The meals included insect larvae (*Tenebrio molitor*), fruits, eggs, and food pellets. Once a week, three animals were subcutaneously injected with 200 μl of cell culture supernatant containing 0.75 × 10^4^ DENV3 viral copies/ml, followed 24 h later by a subcutaneous injection of an equal volume of anti-DENV2 hyperimmune serum (1:32 dilution). Control animals received either a subcutaneous injection of 0.2 ml of diluted anti-DENV2 hyperimmune serum (1:32) once a week (*n* = 2) or were not injected (*n* = 4). All of the animals included in the study were euthanized after 12 weeks to perform tissues analysis. *C. penicillata* is a small (13 cm high, 344 g body weight) New World primate. We selected nine individuals (body weight between 230 and 400 g) feed with insect larvae (*Tenebrio molitor*), eggs, fruits and food pellets. They had free access to water and were fed once or twice a day. Two hundred microliters of cell culture infected supernatant were subcutaneously injected once a week in three animals. These injections contained 0.75 × 10^4^ DENV3 viral copies/ml were followed 24 h later by another subcutaneous injection of equal volume of diluted anti-DENV2 hyperimmune serum (1:32). Control subjects received either equal volume and dilution of anti-DENV2 hyperimmune serum (*n* = 2) or were not injected (*n* = 4).

After 12 weeks all subjects were euthanized to perform tissue analyses.

### Histology and immunohistochemistry

After an overdose of 1:3 xylazine (20 mg/ml) and ketamine (50 mg/ml), all animals were transcardially perfused with heparinized saline followed by 4% paraformaldehyde in 0.1 M phosphate buffer (pH 7.2–7.4). Brains were cut using a vibratome (80 μm thickness) and processed for selective microglia immunolabeling. For immunolabeling, free-floating sections were pretreated with 0.2 M boric acid (pH 9) at 65–70°C for 60 min to improve antigen retrieval. Then sections were washed in 5% phosphate-buffered saline (PBS), immersed for 20 min in 10% normal goat serum (Vector Laboratories), and incubated with rabbit anti-IBA-1 (Wako Chemicals, USA Inc.) (2 μg/ml diluted in 0.1 M PBS; pH 7.2–7.4) for 3 days at 4°C with gentle, continuous agitation. After washing, sections were incubated overnight with a biotinylated secondary antibody (goat anti-rabbit for IBA-1, dilution 1:250 in PBS; Vector Laboratories). We inactivated endogenous peroxidases by immersing the sections in 3% H_2_O_2_ in PBS, then washed the sections in PBS and transferred them to a solution of avidin-biotin-peroxidase complex (VECTASTAIN ABC kit; Vector Laboratories) for 1 h. The sections were washed again before incubation in 0.1 M acetate buffer (pH 6.0) for 3 min. Finally, sections were developed in a solution of 0.6 mg/ml diaminobenzidine, 2.5 mg/ml ammonium nickel chloride, and 0.1 mg/ml glucose oxidase (Shu et al., 1988). We confirmed the specificity of the immunohistochemical pattern by omitting the primary antibody (Saper and Sawchenko, 2003). This negative control resulted in the absence of immunolabeling in all structures.

### Viral RNA isolation and reverse transcription PCR

The viral RNA used for reverse transcription PCR (RT-PCR) was extracted from serum samples by the PureLink RNA Mini Kit (Ambion, Austin, Texas, USA) following the manufacturer's protocol and quantified using a Qubit 2.0 fluorometer (Invitrogen, Carlsbad, CA, USA) with a Qubit RNA BR Assay kit (Invitrogen) following the manufacturer's protocol. Afterwards, viral RNA was used to synthesize cDNA using the EXPRESS One-Step Superscript qRT-PCR Universal kit (Invitrogen) with primers as described elsewhere (Li et al., 2010).

### Viral load determination by quantitative real-time PCR (qPCR)

For the quantification of viral load, a standard curve was constructed using a plasmid provided by cloning of the amplicon using the TOPO TA Cloning Kit (Invitrogen) according to the manufacturer's protocols. Serotype-specific DENV3 primers (DENV-3F, GGACTGGACACACGCACTCA and DENV-3C, CATGTCTCTACCTTCTCGACTTGTCT) were used to create amplicons. Competent bacterial cells (*Escherichia coli* strain TOP10F) were previously prepared by the calcium chloride method (Aich et al., 2012). Plasmid DNA was extracted with a Miniprep DNA Purification System kit (Promega Corporation, Madison, Wisconsin, USA) following the manufacturer's protocol. The concentration of recombinant plasmids containing DENV3 inserts was determined with a Qubit 2.0 fluorometer (Invitrogen) with a Qubit dsDNA BR Assay kit (Invitrogen) following the manufacturer's protocol. Afterwards, the clone was transcribed into RNA using Megascript Transcription T7 (Ambion) according to the manufacturer's protocol. The primer pairs used for generating amplicons were the same utilized to reverse transcription using the same protocol and commercial kits as above described. cDNA quantification were processed by serial dilution of the generated amplicon.

TaqMan quantitative real-time RT-PCR (RT-qPCR) was also used during the assay. RT-qPCR was performed with the ABI Prism 7500 Sequence Detection System (Applied Biosystems, Foster City, CA, USA) with thermal cycling conditions set as follows: one cycle at 50°C for 2 min, followed by 45 cycles at 95°C for 10 min, 95°C for 15 s, and 56°C for 1 min. DENV3 viral load is expressed as viral particles/mL, based on the standard curve constructed using serial dilutions of plasmids containing the DENV3 insert at concentrations ranging from 8 × 10^1^ to 8 × 10^7^ viral particles/mL.

### Photomicrographic documentation and three-dimensional reconstruction

Digital photomicrographs were taken with a digital camera (Microfire; Optronics, Fremont, CA, USA) coupled to a Nikon microscope (Optiphot-2; Melville, NY, USA). Digital photomicrographs were processed with Adobe Photoshop 13.0 C.S.6 software (San Jose, CA, USA) for scaling and for adjusting the levels of brightness and contrast, which were applied to the whole image. The selected micrographs display representative hippocampal sections from each experimental group. Microglial cells were digitally reconstructed in three dimensions (3D) with the aid of a NIKON Eclipse 80i microscope (Tokyo Japan) equipped with a motorized stage (MAC200; Ludl Electronic Products, Hawthorne, NY, USA). Images were acquired under oil immersion with a high-resolution, 100 × oil immersion lens, a plan fluorite objective (Nikon, numerical aperture 1.3, depth of field = 0.19 μm), and a computer with Neurolucida software (MBF Bioscience Inc., Frederick, MD, USA). All cells with dendritic trees that were unequivocally complete were included for reconstruction (cells were discarded when branches seemed to be artificially cut or were not fully IBA-1 immunolabeled). Terminal branches were typically thin. Cells were systematically and randomly selected from polymorphic layer of the dentate gyrus. In the chosen sections, the margins of the polymorphic layer of the dentate gyrus were clearly distinguished from the adjacent layers.

## Results

A reduction in body weight (14 and 20%) in two of the three infected subjects was observed and all of the infected animals were less active and showed hair losses (Videos S1, S2), clinical signs consistent with virally-induced sickness behaviors; no other overt changes were detectable. In infected animals, 1.0–1.46 × 10^6^ viral particles/ml were detected in the serum.

**Figure 1:**
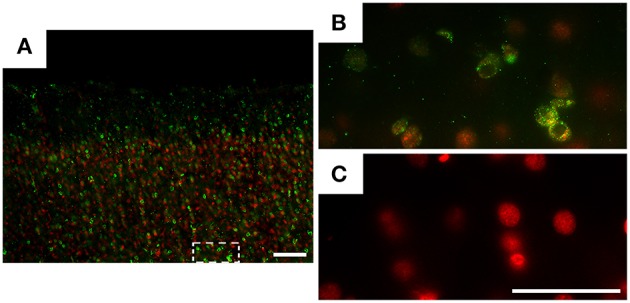
**Photomicrographs of parasagittal parietal cortex sections from infected (A,B) and control (C) animals, submitted to indirect immunofluorescence for DENV3 virus antigens (green)**. DNA counterstaining was done with ethidium bromide (red). Immunofluorescence for virus antigens (green dots) was found only in infected monkeys **(A,B)**. Rectangular dotted line area **(A)** is shown in high power **(B)**. Control subject did not show virus antigens immunolabeling **(C)**. Sections from both infected and control subjects were taken from lateral parietal cortex of *Callithrix penicillata*. Gross morphologies of parasagittal illustrated sections were close to 5.1 mm lateral to the interhemispheric fissure. Details of the stereotaxic atlas of *Callithrix jacchus* can be found in: http://www.ncbi.nlm.nih.gov/books/NBK55676/figure/parasagittal_plane_08.x1/?report=objectonly. Scale bars **(A)**: 100 μm, **(B)**: 50 μm and **(C)**: 50 μm.

Figure 1 shows immunolabeled brain sections for DENV3 virus antigens, counterstained with ethidium bromide. Note that immunofluorescence for virus antigens was only found in infected monkeys (Figures 1A,B). Control subjects did not show immunolabeling for virus antigens (Figure 1C).

**Figure 2:**
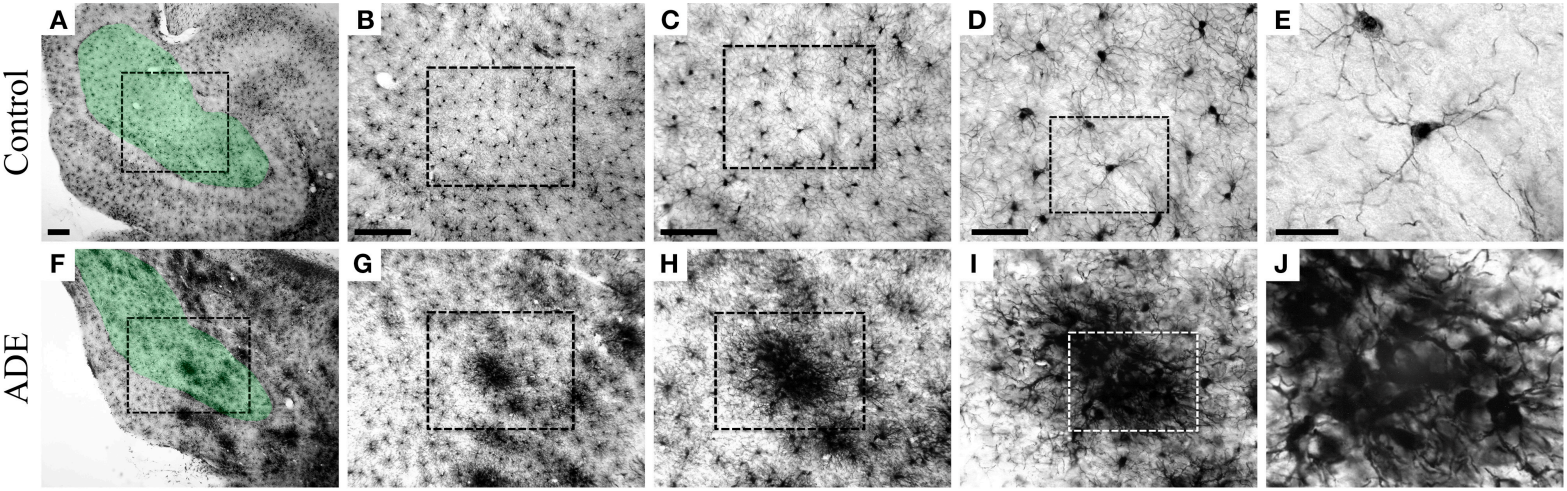
**Low- and high-power pictures from IBA-1 hippocampal immunolabeled sections to illustrate microglia from the polymorphic layer of the dentate gyrus**. Low power pictures: boundaries of areas of interest. High power: morphological features of microglia in various magnifications to illustrate microglia from control **(A–E)** and clustered microglia from infected **(F–J)** monkeys. From left to right scale bars correspond to: 200, 200, 100, 50, and 20 μm.

Individual microglia were selected for comparative 3D morphometric analysis using stereological unbiased sample approach. Randomized and systematic samples (West, 1999) were taken from the polymorphic layer of the dentate gyrus of both ADE-infected and uninfected monkeys. Unlike non-ADE animals, the dentate gyrus (DG)-microglia were morphologically activated and displayed significantly larger somas, thicker primary branches, and more ramified distal branches compared to control animals. Furthermore, throughout the CNS of ADE-infected monkeys, the microglia were often clustered, with a seemingly random distribution, in the parenchyma of ADE monkeys. These microglial clusters were of variable size, and contained microglia with larger somas, thicker primary branches, and short intermingled processes with superimposed domains. These intermingled branches made 3D reconstruction very difficult and imprecise, therefore no clustered microglia were reconstructed. The clustered microglia would fit the criteria of stage 3 activation (Figure 2).

**Figure 3:**
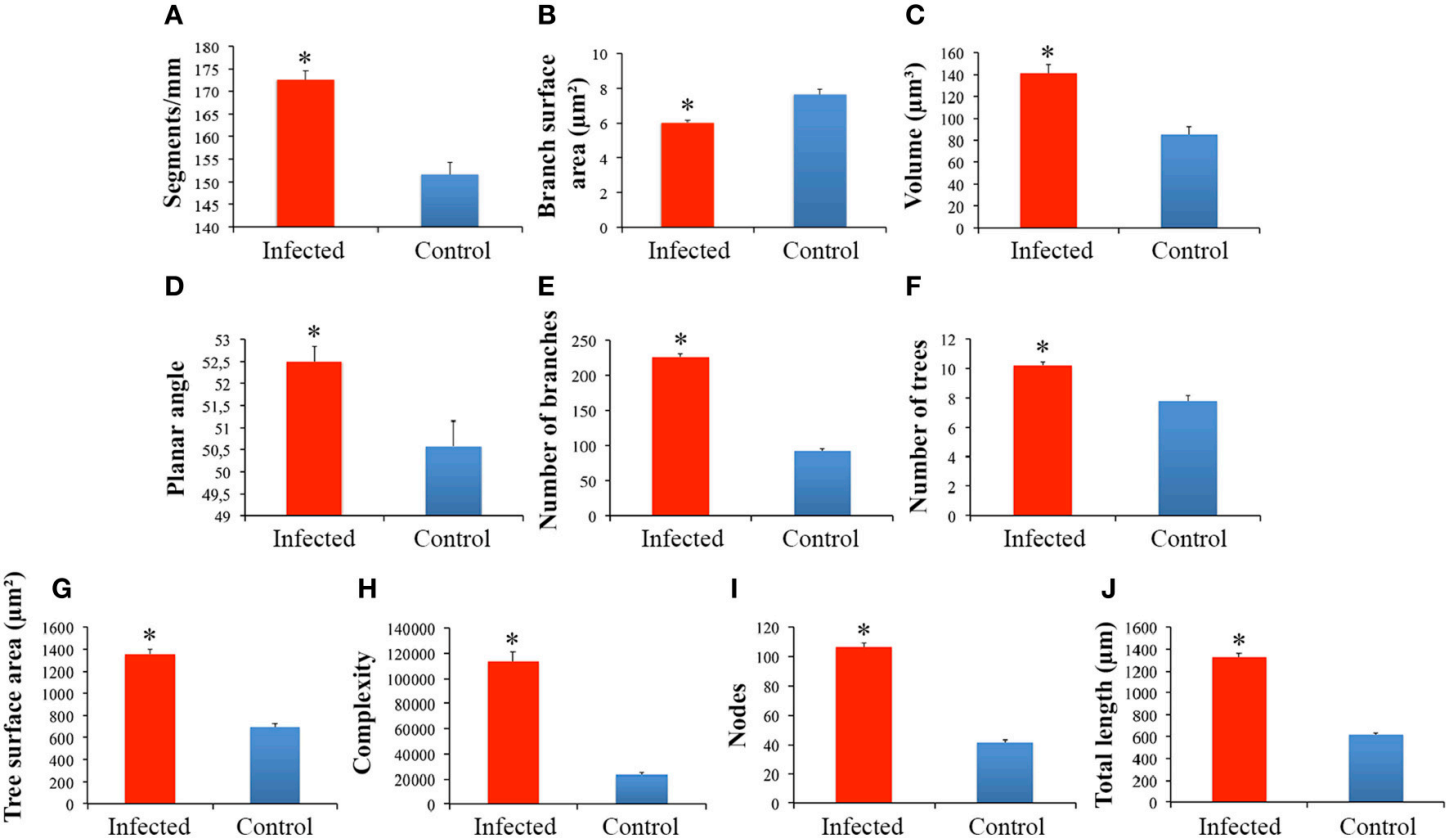
**Graphical representations of the mean and standard errors values of microglia morphological features of branches from infected and control monkeys to illustrate significant differences between microglial processes in the polymorphic layer of dentate gyrus from infected and control animals (A–J)**. (^*^)Indicates statistical significant differences between morphometric features of microglia from infected and control groups.

**Figure 4:**
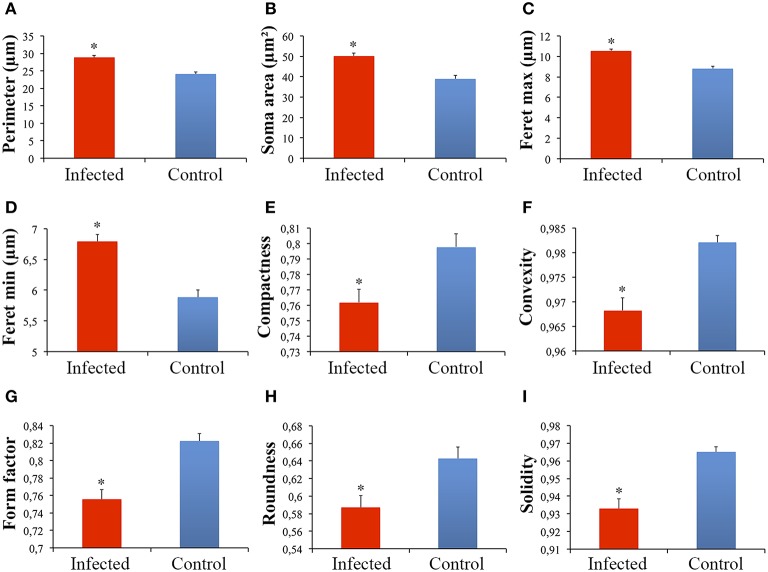
**Graphical representations of the mean and standard errors values of microglial soma morphological features (A–I) in the polymorphic layer of dentate gyrus from infected and control monkeys**. (^*^)Indicates statistical significant differences between morphometric features of microglia from infected and control groups.

Significant differences between microglial morphology from infected and control animals were found in the polymorphic layer of the dentate gyrus (Figures 3A–J, 4A–I). Numerical details of 19 morphological variables showing significant differences in a total of 22 are shown in Table 1.

**Table 1:** **Morphological significant differences between microglia from antibody disease enhanced (ADE) dengue infected monkeys and controls injected with anti-DENV2 antibodies (Anti-DENV2) or naive (NI) monkeys**.

**3-D morphometric variables**	**Mean/± s.e.m**.		**U or two-tail *t*-values**	**Z(U)**	***p*-values**
	**ADE**	**Anti-DENV2/NI**			
Tree total length (μm)	1324.94±34.44	615.15±54.4	156	11.14	0.0001
Number of nodes	106.63±2.56	41.68±1.32	46	11.45	0.0001
Tree surface area (μm^2^)	1354.15±43.09	694.45±32.07	797	9.31	0.0001
Number of trees	10.21±0.25	7.79±0.40	1657	6.85	0.0001
Number of segments/mm	172.60±1.98	151.69±2.49	1921	6.09	0.0001
Number of branches	226.32±5.13	92.13±2.68	37	11.48	0.0001
Branch volume (μm^3^)	141.41±7.29	84.95±7.04	*t* = 5.5		*p* < 0.0001
Branch surface area (μm^2^)	6±0.15	7.63±0.32	2855	3.42	0.0006
Planar angle (°)	52.50±0.33	50.56±0.58	2976	3.07	0.0021
Complexity	113.404±7850	23.342±1798	444	10.32	0.0001
Soma perimeter (μm)	28.86±0.50	24.11±0.59	6.11		0.0001
Soma area (μm^2^)	50.11±1.46	38.91±1.67	*t* = 5.04		*p* < 0.0001
Feret max (μm)	10.54±0.20	8.81±0.25	*t* = 5.39		*p* < 0.0001
Feret min (μm)	6.79±0.11	5.89±0.12	*t* = 5.47		*p* < 0.0001
Convexity	0.97±0.003	0.98±0.001	2706	3.84	0.0001
Form factor	0.59±0.01	0.76±0.01	2449	4.58	0.0001
Solidity	0.93±0.005	0.96±0.003	2382	4.77	0.0001
Roundness	0.59±0.01	0.64±0.01	*t* = −2.95		*p* < 0.004
Compactness	0.76±0.009	0.80±0.009	*t* = 2.93		*p* < 0.004

**Figure 5:**
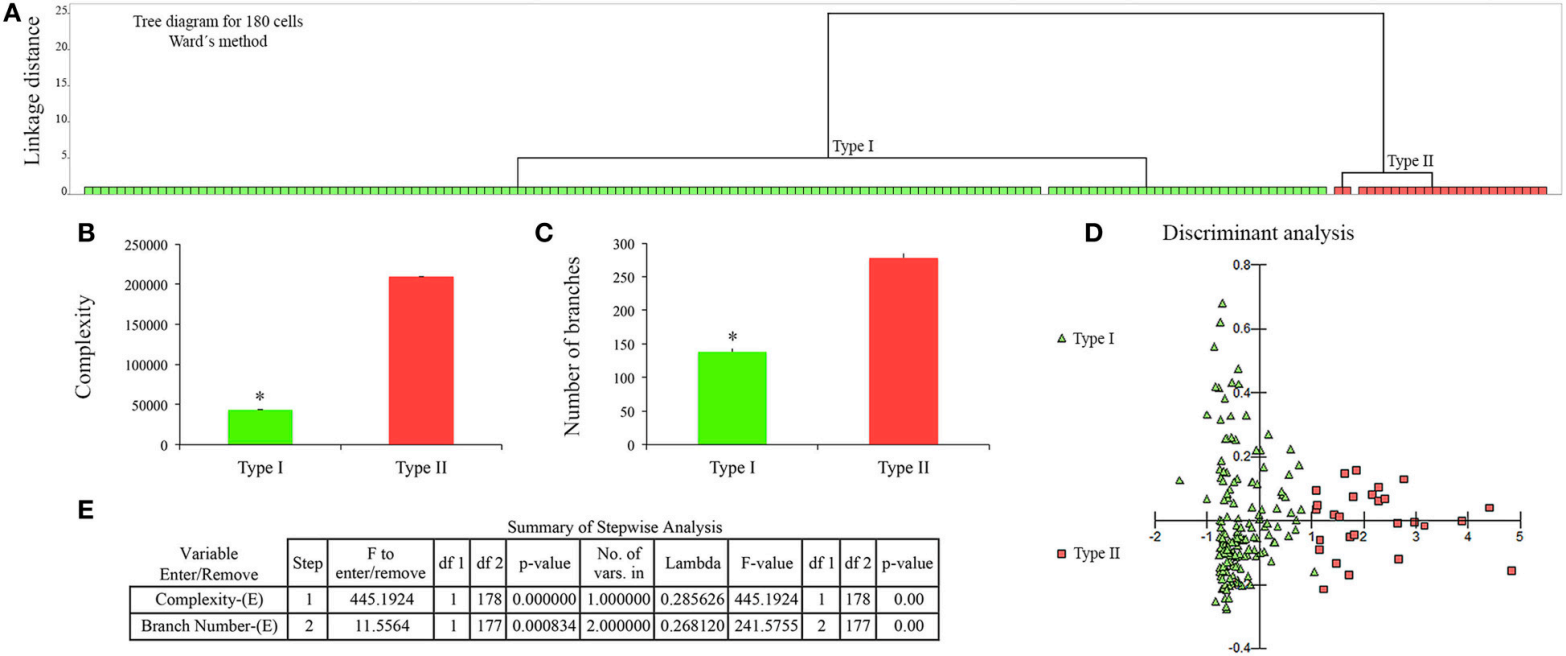
**Cluster and discriminant analysis of 180 microglial cells from control (*n* = 90) and infected (*n* = 90) monkeys**. **(A)** Dendrogram to illustrate the results of cluster analysis. X and Y axes correspond to type of microglia and linkage distance between clusters, respectively. Note complete segregation of Type I (green) and Type II (red) microglia in two distinct clusters. **(B,C)** Graphic representations of mean and standard errors of the two variables that most contributed to cluster formation to illustrate statistical significant differences (^*^) = two tail *t*-tests, *p* < 0.0001 between morphometric features of type I and Type II microglia. **(D)** Graphic representation of discriminant analysis; green triangles corresponds to Type I and red squares to Type II microglia. **(E)** Summary of the step forward discriminant analysis results.

To understand whether the changes reflect discrete or continuous alterations in morphology we used statistical analysis described elsewhere (Yamada and Jinno, 2013). To that end we estimated the multimodality index (MI) based on skewness and kurtosis of our sample for each morphometric variable as previously defined elsewhere: MI = [M3 + 1]/[M4 + 3], where M3 is skewness and M4 is kurtosis and n is sample size (Kolb et al., 1994; Schweitzer and Renehan, 1997). Kurtosis and skewness describe the shape of the data distribution and enable to distinguish between unimodal, bimodal or multimodal curves. Multimodal data sets are essential for separating a population of cells into cell types (Schweitzer and Renehan, 1997). We found that a few microglial morphological features showed a multimodality index >0.55 and this index value indicates that the distribution is at least bimodal and may be multimodal, and these particular features were selected for cluster analysis as previously described (Schweitzer and Renehan, 1997). Thus, our cluster analysis included the following morphological features: complexity, number of branches, branch surface area and soma convexity and solidity. We used the Ward's method with standardized variables, square Euclidian distances and a tree diagram (dendrogram) to illustrate the classification generated by cluster analysis (Figure 5). To investigate differences among groups generated by cluster analysis and discard variables which are little related to group distinction, we used discriminant analysis. Discriminant analysis derives an equation as linear combination of the independent variables (quantitative morphometric features) that will discriminate best between the groups (qualitative microglial morphological phenotypes). Detailed information about discriminant analysis can be found in: https://www.researchgate.net/file.PostFileLoader.html?id=54eb12afef97130f298b4576&assetKey=AS%3A273713604300800%401442269816239.

This procedure revealed that complexity was by far the morphometric feature that most contributed to cluster formation (Figure 5). From hierarchical cluster analysis we categorized microglia into two groups designated I and II. Type I and II cell clusters showed cell morphologies quite distinct one from another (Table 1). We designated as type I the microglia which exhibited processes with significantly smaller values of complexity and number of branches as compared to type II. As compared to type II, type I microglia also showed arbors with fewer nodes, a lower density of segments/mm, smaller branch volumes, branch angles and tree surface area and shorter total branch length. Type I microglia had smaller soma perimeters and Feret min, but higher convexity and solidity than type II microglia (Table 1).

Taken together these findings suggest that, at least in the ADE primate model of dengue infection, microglial changes between type I and II are not a morphological continuum. Indeed, it seems from multivariate statistical analysis that type I microglia may change to type II without undergoing intermediate morphological stages.

**Figure 6:**
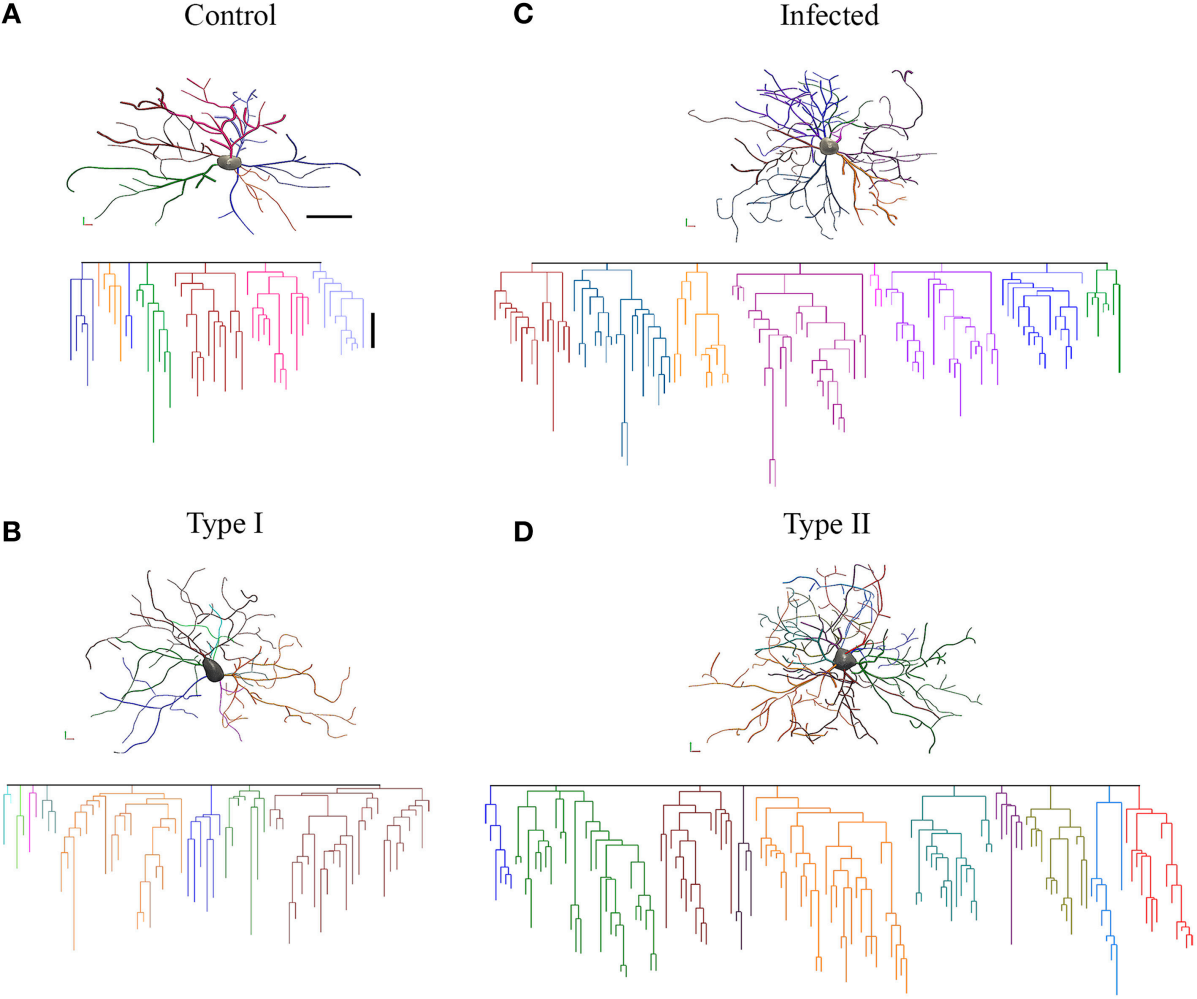
**Three-dimensional reconstructions of selected microglia from the polymorphic layer of the dentate gyrus from control (A), Type I microglia (B), infected monkey (C), and Type II (D) microglia**. Microglia three-dimensional reconstructions depicted here exhibit morphometric features representative of the mean morphological features of other microglia within the relevant experimental group. Individual branches were distinctively colored to facilitate examination. Linear dendrograms of microglial arbors are shown below each 3D reconstruction. The length of each branch segment is displayed to scale; sister branches are horizontally displaced. Branch colors correspond to the 3D reconstructions above. Dendrograms were plotted and analyzed with Neuroexplorer (MicroBrightField). Note close similarity between control and type I microglia and between infected and type II microglia. Scale bars 3-D = 15 μm; dendrogram = 10 μm.

It is readily evident that compared to control monkeys, microglia from ADE-infected monkeys seem to be morphologically closer to type II and microglia from control monkeys to type I. Dendrograms of the 3D reconstructions are particularly useful for visualizing the differences in the number of processes of type I and type II, and between control and infected monkey microglia (Figure 6).

Finally, we estimated the percentages of type I and type II microglia present in control and infected monkey samples and found that 27 of 90 microglia (30%) from the polymorphic layer of the dentate gyrus of infected monkeys were classified as type II. No type II cells were detected in control monkeys.

**Figure 7:**
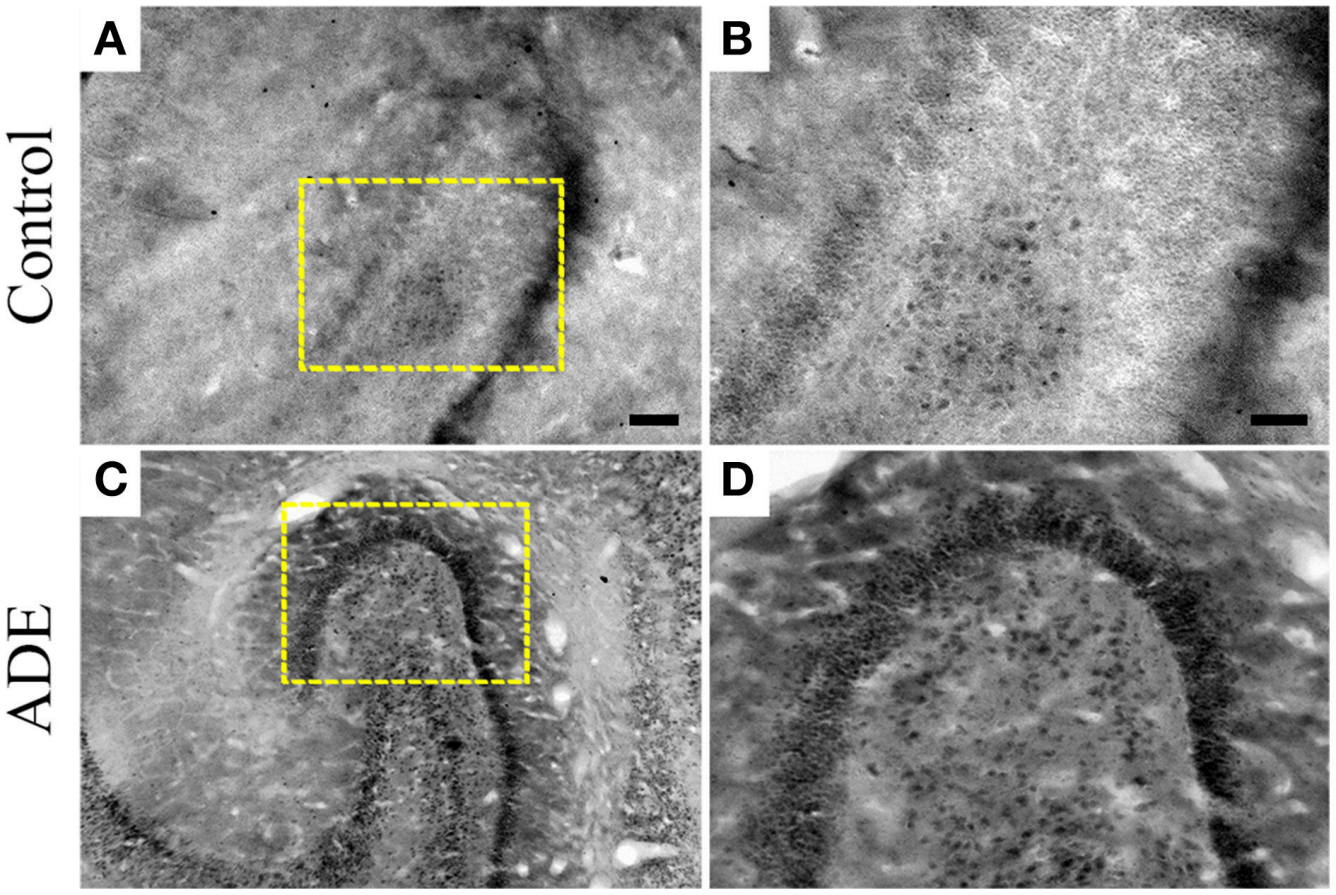
**TNFα immunolabeling in selected sections of dentate gyrus from both control (A,B) and antibody-enhanced dengue disease (C,D) monkeys**. Scale bars: 250 μm.

The significant increase in the number of processes in type II microglia observed in infected monkeys was found to be associated with conspicuous increase in TNFα immunolabeling in the polymorphic layer of dentate gyrus (Figure 7).

## Discussion

In a previous report, we translated the ADE model to *C. penicillata*. The infected monkeys exhibited microglial changes, including hyper-ramification (stage 2 activation) and clustering (stage 3 activation). Infected monkeys also exhibited DENV3 viral antigens in multiple CNS areas (Vasconcelos et al., 2016). Here, we used hierarchical cluster and discriminant analyses to evaluate and classify morphological features observed in 3D reconstructions of microglia from ADE animals. Based on the 3D reconstructions, we found two distinct morphological phenotypes among microglia: type II microglia were detected exclusively in infected monkeys; type I microglia were detected in both control and infected monkeys. A third type of microglia, also exclusively detected in infected monkeys, was not reconstructed, due to the intermingling of superimposed branches, which made it very difficult to build precise 3D reconstructions. This type of microglia was found in groups of various sizes, in many CNS areas. The groups of cells with superimposed domains appeared to be in stage 3 activation, and their clustering behavior was suggestive of microglial mobilization to damaged areas.

In the adult brain, damage can induce a variety of morphological changes in microglia that range between a multiple-process/highly-ramified morphology and a rounded/amoeboid morphology. These morphologies may represent extremes of a continuum that spans multiple intermediate stages (Karperien et al., 2013; see Harry and Kraft, 2012) for a review. Previous studies have described a microglial transition from a ramified resting state to an amoeboid state, with intermediate states of hypertrophy and a bushy appearance. That transition was associated with increasing cell motility and proliferative activity. However, for morphological cell changes that appear to be continuous (i.e., when microglia transition from stage 1 to stage 2), it may be essential to use an unbiased method to quantify fine anatomical details. In the present study, we aimed to detect and quantify details along this continuum of morphological possibilities. Therefore, we used a stereological, random, systematic sample approach, combined with 3D reconstructions of microglia, and hierarchical cluster and discriminant analyses, as described elsewhere. Our findings showed that microglia could be separated into two principal groups, designated type I and type II. Type II was only found in infected monkeys; thus, the control group had only type I microglia.

Microscopic 3D reconstructions may be affected by mechanical factors associated with vibratome sectioning and/or dehydration procedures. These methodological limitations imposed constraints that must be taken into consideration when interpreting the results of the present study (Hosseini-Sharifabad and Nyengaard, 2007). Indeed, it has been demonstrated that, in the z-axis (perpendicular to the cutting surface), sections shrink by ~75% of the cut thickness after dehydration and clearing procedures (Carlo and Stevens, 2011). However, this shrinkage is not linear; therefore, we did not make corrections to the z-axis or to the X/Y axes in the microglial reconstructions of the present report.

The criteria we used in selecting individual microglial cells for 3D reconstruction were systematically blinded and randomized. Also, the number of elements selected for reconstruction was rather large (180 in total, 90 in each group). Therefore, it is reasonable to assume that no *a priori* sample bias was introduced by the choice of objects of interest among subjects. Although the evidence in the present report was indirect and the study results were explicitly correlational, our findings provided the opportunity to formulate hypotheses about the relationships between microglial morphological changes and cytopathic virus infections. Indeed, our findings suggested that, for the ADE dengue infection model in primates, microglial activation—contrary to previous reports—was discontinuous in nature, and the pattern of activation followed a step function. This finding may prove to be generalizable to other pathologies that involve microglia activation.

In a previous report, among nine morphometric features of microglia, only two parameters exhibited a multimodality index larger than 0.55 (Soltys et al., 2005). Those authors suggested that a principal component analysis was an effective tool for investigating microglial morphological responses to transient global ischemia. They showed that the first two principal components could explain more than 73% of the observed variability. Thus, they concluded that those two components may be sufficient to describe the morphological diversity of the cells and to determine the dynamics and direction of the changes. However, because only two morphometric variables fulfilled the criteria of MMI >0.55 in that study, the authors did not apply a cluster analysis. In the present study, five morphometric variables fulfilled the MMI criteria; thus, we could apply both a principal component (PC) analysis and a cluster analysis, as previously suggested (Yamada and Jinno, 2013). We found that PC1 and PC2 explained 79% of the observed variability, which validated our cluster analysis findings (Yamada and Jinno, 2013). Because all principal components are reciprocally orthogonal, it is reasonable to assume that each component described a different aspect of microglial morphology (Soltys et al., 2005). With an effective cell typing strategy, each cell type (cluster of cells) should have characteristics that distinguish it from all other cell types (Schweitzer and Renehan, 1997). Indeed, in the present report, subsequent two-tailed *t*-tests also indicated that type I microglia, on average, differed significantly from type II microglia, in 12 of 22 morphometric features. However, we did not reconstruct microglia from clusters of cells with the most intense morphological changes; thus, it is likely that we missed some of the more severe microglial morphological changes in our sample.

Previously, it was suggested that qualitative assessments of microglia morphology could not reliably predict function (Boche et al., 2013; Santos-Filho et al., 2014; Taylor et al., 2014; Clark et al., 2015; Gomez-Nicola and Perry, 2015). However, recent studies have demonstrated that the form-function model is a good starting point for investigating the influence of multivariate factors that affect microglial morphology and function (Nimmerjahn et al., 2005; Wake et al., 2009; Karperien et al., 2013). In the present report, we explored the influence of an ADE-mediated dengue infection on the morphology of microglia in the polymorphic layer of the dentate gyrus. We found that the dengue infection caused increases in the number and volume of branches, the number of nodes, the density of segments, the tree surface area, the planar angle, the total length, and the complexity of microglia. From the branch analysis, complexity appeared to be the morphological feature that contributed most to the cluster analysis results. Complexity was defined previously (Pillai et al., 2012) with the following equation:

$$\begin{array}{l} \text{Complexity}=\\ \;\left[\text{Sum of the terminal orders}+\text{Number of terminals}\right]\\ \;\times \;\left[\text{Total branch length}∕\text{Number of primary branches}\right]. \end{array}$$

For details, see the Neurolucida Explorer website: Branched Structure Analysis: Neuron Summary (http://www.mbfbioscience.com/help/nx11/Default.htm#Analyses/BranchedStructure/neuronSumm.htm). From this equation, which was previously applied to neuronal morphology, high complexity values reflect more ramification and longer microglial processes. This index of microglial morphological complexity was associated with hyper-ramified microglia, observed in the absence of inflammation or neurodegeneration (Hinwood et al., 2012), in the prefrontal cortex of rats submitted to chronic stress. Although chronic stress significantly increased the internal complexity of microglia, it enhanced ramification without altering the area occupied by the cell. It is noteworthy that mice subjected to chronic stress expressed TNFα, albeit at relatively low levels, and they also contained activated and proliferating microglia in the prefrontal cortex (Couch et al., 2013). However, in the present study, we observed a pattern that was closer to a pattern previously demonstrated in rats submitted to a lipopolysaccharide challenge (Kloss et al., 2001) or to a viral challenge (de Sousa et al., 2015), where an increased inflammatory response was associated with a conspicuous pro-ramifying effect in microglia. It was previously suggested that an increase in the expression of β1-integrin (CD29) after a lipopolysaccharide injection could cause alterations in microglial branches. That hypothesis was supported by the observation that, after lipopolysaccharide exposure, integrin immunoreactivity increased in ramified microglia in the mouse brain (Kloss et al., 2001). The changes we observed in the microglia of infected monkeys were associated with an increase in TNFα immunolabeling, which suggested that these cells might be phenotypically classified as M1-like cells. Therefore, we consider it reasonable to suggest that the highly branched microglia frequently found in infected animals may indicate that, in response to the infection, the microglia underwent an early stage of activation. We also suggest that the clustered distribution of activated microglia may indicate that some activated cells migrated to injured sites in the parenchyma, as previously described *in vitro* (Heppner et al., 1998) and *in vivo* (Ullrich et al., 2001). In the present study, very few rounded cells were visible, but their appearance, clustering behavior, and cytokine profiles suggested that these cells may be motile and may contribute to CNS injury. Future studies should explore the association of the microglial activation with markers and the levels of CNS integrity.

## Author contributions

DD, GS, TN, TF, SA, JD, and MS did the experimental procedures including inoculations, immunohistochemistry, and video-recording analysis. DD did the microglial reconstructions; DD, JD, LD analyzed data, collected, and edited microscope images; DD, MS, CD, PD, and CP design the experiments and analyzed the data; DA, PD, CP analyzed data and wrote the manuscript.

## Funding

This study had financial support from CNPq (grant no. 300460/2005-8, 301955/2007-7, 200065/2014-9, and 471444/2006-5), INCT-FHV/CNPq/CAPES/FAPESPA (grant no. 573739/2008-0), FINEP/FADESP (grant no. 01.04.0043.00).

### Conflict of interest statement

The authors declare that the research was conducted in the absence of any commercial or financial relationships that could be construed as a potential conflict of interest.
